# Why do smokers try to quit without medication or counselling? A qualitative study with ex-smokers

**DOI:** 10.1136/bmjopen-2014-007301

**Published:** 2015-04-30

**Authors:** Andrea L Smith, Stacy M Carter, Simon Chapman, Sally M Dunlop, Becky Freeman

**Affiliations:** 1 Centre for Values, Ethics and the Law in Medicine, School of Public Health, University of Sydney, Sydney, New South Wales, Australia; 2 School of Public Health, University of Sydney, Sydney, New South Wales, Australia; 3 Cancer Screening and Prevention, Cancer Institute NSW, Eveleigh, New South Wales, Australia; 4 Prevention Research Collaboration, School of Public Health, University of Sydney, Sydney, New South Wales, Australia

**Keywords:** QUALITATIVE RESEARCH, PUBLIC HEALTH, PREVENTIVE MEDICINE, PRIMARY CARE

## Abstract

**Objective:**

When tobacco smokers quit, between half and two-thirds quit unassisted: that is, they do not consult their general practitioner (GP), use pharmacotherapy (nicotine-replacement therapy, bupropion or varenicline), or phone a quitline. We sought to understand why smokers quit unassisted.

**Design:**

Qualitative grounded theory study (in-depth interviews, theoretical sampling, concurrent data collection and data analysis).

**Participants:**

21 Australian adult ex-smokers (aged 28–68 years; 9 males and 12 females) who quit unassisted within the past 6 months to 2 years. 12 participants had previous experience of using assistance to quit; 9 had never previously used assistance.

**Setting:**

Community, Australia.

**Results:**

Along with previously identified barriers to use of cessation assistance (cost, access, lack of awareness or knowledge of assistance, including misperceptions about effectiveness or safety), our study produced new explanations of why smokers quit unassisted: (1) they prioritise lay knowledge gained directly from personal experiences and indirectly from others over professional or theoretical knowledge; (2) their evaluation of the costs and benefits of quitting unassisted versus those of using assistance favours quitting unassisted; (3) they believe quitting is their personal responsibility; and (4) they perceive quitting unassisted to be the ‘right’ or ‘better’ choice in terms of how this relates to their own self-identity or self-image. Deep-rooted personal and societal values such as independence, strength, autonomy and self-control appear to be influencing smokers’ beliefs and decisions about quitting.

**Conclusions:**

The reasons for smokers’ rejection of the conventional medical model for smoking cessation are complex and go beyond modifiable or correctable problems relating to misperceptions or treatment barriers. These findings suggest that GPs could recognise and respect smokers’ reasons for rejecting assistance, validate and approve their choices, and modify brief interventions to support their preference for quitting unassisted, where preferred. Further research and translation may assist in developing such strategies for use in practice.

**Strengths and limitations of this study**

- The qualitative design allowed us to extend the existing literature on barriers and facilitators of assistance utilisation to provide a more in-depth discussion of the complex reasons of why smokers may choose to quit unassisted.
- Concurrent data collection and analysis allowed interesting, unanticipated findings to be followed up and explored in subsequent interviews.
- Asking ex-smokers to talk about previous assisted and unassisted quit attempts provided new insights into why some smokers go on to quit unassisted.
- As participants were ex-smokers who had quit unassisted between 6 months and 2 years ago, it is possible that their recollections may have been subject to recall bias.

## Introduction

Smoking cessation researchers, advocates and healthcare practitioners have tended to emphasise that the odds of quitting successfully can be increased by using pharmacotherapies such as nicotine-replacement therapy (NRT), bupropion and varenicline^1^ or behavioural support such as advice from a healthcare professional^2–5^ or from a telephone quitline.^6^ However, instead of using one or more of these forms of assistance, it appears most quit attempts are unassisted^7^ and most long-term and recent ex-smokers quit without pharmacological or professional assistance.^8^

Researchers have identified a number of issues relating to the choice to use assistance. They generally conclude that failure to use assistance can be explained by treatment-related issues such as cost and access, and patient-related issues such as lack of awareness or knowledge about assistance, including misperceptions about the effectiveness and safety of pharmacotherapy or concerns about addiction.^9–12^

The policy and practice response to the low uptake of cessation assistance has typically focused on improving awareness of, access to, use of assistance and in particular, pharmacotherapy. NRT, bupropion and varenicline are often provided free-of-charge or heavily subsidised by the government or health insurance companies.^13–15^ NRT is on general sale in pharmacies and supermarkets, and is widely promoted through direct-to-consumer marketing.^16^ ^17^ Clinical practice guidelines in the UK, USA and Australia advise clinicians to recommend NRT to all nicotine-dependent (>10 cigarettes per day) smokers.^18–20^ Specialist stop-smoking clinics, and dedicated telephone and online quit services provide smokers with tailored support and advice.^21–23^ These products and services have not had the population-wide impact that might have been expected from clinical trial results,^16^ ^24^ ^25^ leading some researchers to suggest that patient-related barriers such as misperceptions about effectiveness and safety are a greater impediment than treatment-related barriers.^26^ Little attention, however, has been given to how and why smokers quit unassisted.^8^ ^27^ If we can explain how the process of unassisted quitting comes about and what it is about unassisted quitting that appeals to smokers, we may be better placed to support all smokers to quit, whether or not they wish to use assistance.

We conducted a qualitative study to understand why half to two-thirds of smokers choose to quit unassisted rather than use smoking cessation assistance. Smoking cessation researchers have recently highlighted the importance of gaining the smokers’ perspective^28^ ^29^ and suggested qualitative research might provide the means of doing so.^30^ Although a number of qualitative studies have examined non-use of assistance in at-risk or disadvantaged subpopulations,^31–33^ only a few have looked at smokers in general.^26^ ^34^ Cook-Shimanek *et al*^30^ report that few studies have examined explicit self-reported reasons of why smokers do not use NRT; to our knowledge, none has examined explicit, self-reported reasons of why smokers do not use prescription smoking cessation medications.

A qualitative approach was well suited to the research questions guiding the current study, which were:

(1) What does quitting unassisted mean to smokers?

(2) What factors influence smokers’ decisions to quit unassisted?

In order to contextualise the findings of our qualitative study, we also performed a comprehensive review of the literature on non-use of smoking cessation assistance.

## Methods

### Literature review

**Figure 1:**
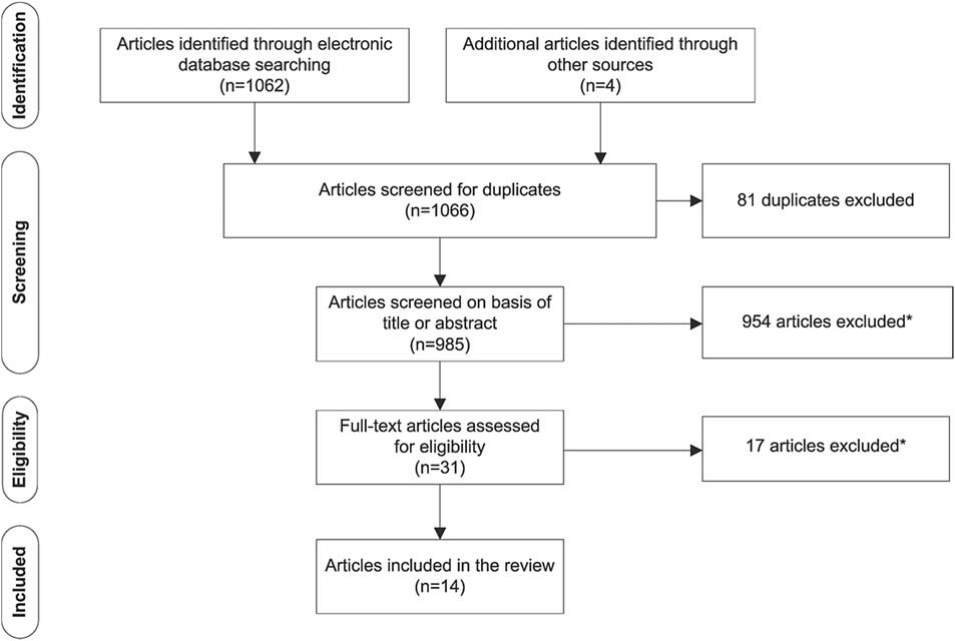
Identification and screening of eligible articles for inclusion in the literature review. *Articles were excluded if they reported only on (1) the characteristics of smokers who did not use assistance; (2) the feasibility/acceptability of a smoking cessation intervention; (3) specific subpopulations, for example, culturally and linguistically diverse populations, pregnant women, or at-risk populations such as hospital patients or youth.

We searched MEDLINE via OvidSP, PsycINFO via OvidSP and CINAHL via EBSCO in February 2015 for articles reporting on non-use of smoking cessation assistance (see online supplementary file 1 for search strategies and results). We complemented this search strategy by manually searching the reference lists of relevant papers. Articles were included if: (1) the article reported on non-use of smoking cessation assistance; (2) the article was published in 2000 or later; and (3) the article was in English. Articles were excluded if (1) they reported only on the characteristics or demographics of smokers who did not use assistance; (2) the study was evaluating the feasibility of a smoking cessation intervention; or (3) the study reported only on specific subpopulations such as pregnant women, youth or prisoners. We identified 1066 articles of which 14 met the inclusion criteria (figure 1). The included papers were not critically appraised for quality as our intent was not to synthesis the results of the studies, but to report on how the issue is currently framed.

### Qualitative study design

A constructivist grounded theory methodology underpinned the study design, research questions, data collection, analysis and interpretation.^35^ In a grounded theory study, data collection and analysis are iterative with each informing the other. Sampling is theoretically driven, that is, researchers shape their sampling strategy based on the developing analysis. Recruitment continues until theoretical saturation has occurred and an explanation generated for the process or phenomenon under investigation.^36^

### Recruitment and participant selection

We recruited from the general community using traditional media (media release, print and online newspaper articles, talk-back radio) as well as social media (Twitter, Facebook). Potential participants were screened for eligibility. Eligible participants were adult (18+ years of age) ex-smokers who had quit unassisted in the previous 6 months to 2 years. Risk of relapse to smoking, which reduces with time quit,^37^ ^38^ was balanced against potential for recall bias.^39^ Participants’ smoking and quitting histories (eg, cigarettes per day, years smoking, number and type of prior quit attempts) and basic demographic information (eg, age, gender, education, income and geographical location) were collected. Eligible participants were initially purposively sampled (n=9), and then theoretically sampled on the basis of their screening information (n=12). We selected ex-smokers with varied smoking and quitting histories from a diverse range of backgrounds. This sampling strategy ensured we generated rich, relevant and diverse data pertinent to the research questions and to our evolving theories about quitting and use/non-use of assistance. Participants were offered AU$80 reimbursement for sparing their time. We interviewed 21 ex-smokers who had quit without assistance within the past 6 months to 2 years. Participant characteristics are summarised in table 1.

**Table 1:** Participant characteristics

Characteristic	Participants (n=21)
Gender	
Male	9
Female	12
Age (years)	
20–29	1
30–39	5
40–49	3
50–59	8
60–69	4
Geographical location*	
Major cities	13
Inner regional Australia	2
Outer regional Australia	6
Remote Australia	0
Very remote Australia	0
Total household income (before tax) (AU$)†	
≤30K	4
>30–60K	3
>60–90K	3
>90–120K	6
>120K	4
Experience of assistance	
Had never tried to quit before	2
Had never used assistance to quit	7
Had previously used assistance to quit	12
Previous quit attempts	
None	2
<3	10
3–10	7
>10	2
Recruitment method‡	
Traditional	12
Social media	9
Interview format	
Face-to-face	8
Telephone	13

*Classified according to the Australian Standard Geographical Classification Remoteness Area system.

†One participant did not answer the question on income.

‡Traditional: media release, print and online newspaper articles, talk-back radio; social media: Twitter, Facebook.

### Conducting the interviews

Interviews took place between December 2012 and December 2013. Where geographically feasible, participants were encouraged to be interviewed face-to-face; however, the final decision was left to the participant. All interviews were conducted by ALS. The University of Sydney Human Research Ethics Committee approved all study procedures and materials. Potential participants were provided with a participant information sheet; participants provided written consent for their participation prior to enrolment in the study.

A semistructured interview guide was used for each interview, but the specific questions asked reflected the quitting experiences of the participant and the stage in data collection. Questions evolved as recruitment and interviewing progressed, with subsequent interviews becoming more specific in order to help the development of provisional ideas and theories. Both the screening questionnaire and interview guide were pilot tested prior to start of the study.

### Data capture, coding and analysis

Interviews were audio recorded and transcribed verbatim; interviews lasted between 37 min and 1 h 50 min. Field notes were made directly after each interview. Theoretical saturation was reached after 21 interviews; at this point our evolving ideas and theories were fully evidenced from the data, and few or no new insights were forthcoming from participants.

Data management and analysis were aided by use of computer-assisted qualitative data analysis software NVivo 10 (QSR International). Data analysis involved (1) using the first 5 interview transcripts and field notes to create detailed codes reflecting what appeared to be most important to those participants; (2) sorting the codes into a coding hierarchy; (3) coding the subsequent transcripts, and revising the codes and coding hierarchy as necessary; (4) comparing and contrasting data from within and between interviews; and (5) writing memos. During memoing, the researcher documented the analytical thinking driving the coding process and explored relationships between categories.

Coding and memoing were performed by ALS. The codes, coding hierarchy, memos and evolving ideas and theories were regularly discussed with the other researchers. In addition to experience in tobacco control, each of the researchers had expertise in different areas relevant to the project, including smoking cessation, behavioural psychology, bioethics and qualitative health research methodology. The diversity of viewpoints and experiences were critical to the interpretation of the data.

When the researchers had established the central categories in the analysis, these were mapped against what had been reported in the existing literature. Those categories that had not previously been discussed in the literature were analysed further and connections between them explored.

## Results

### Study perspective

Key categories identified in our data were mapped against reasons for non-use of assistance as reported in the smoking cessation literature (table 2). These included treatment-related and patient-related issues, as well as a number of social–environmental issues. We were encouraged by the consistency between our categories and the findings of previous research.

**Table 2:** Data from current study mapped against known issues related to non-use of smoking cessation assistance

Studies	Issues related to non-use of smoking cessation assistance								
	Treatment-related issues		Patient-related issues						Social**-**environmental issues
	Cost	Access (eg, delay in getting prescription; GP as gatekeeper)	Lack of awareness or knowledge about assistance (including misperceptions):			Overconfidence in own abilities	Not regarded as appropriate:		Social norms (eg, relating to use of assistance or perception of assistance users)
			Effectiveness/ how assistance works	Safety/side effects/future health/new addiction	Availability (eg, how to get NRT, free or subsidised pharmacotherapy or behavioural support)		For the smoker (eg, not addicted enough; do not like using medications)	For quitting (eg, deals with addiction not behavioural/psychological aspects)	
Quantitative									
Etter and Perneger 9				✓		✓			
Bansal *et al* 10			✓	✓		✓			
Cummings *et al* 40			✓	✓					
Hammond *et al* 41			✓			✓			
Mooney *et al* 42			✓	✓					
Gross *et al* 11	✓					✓			
Shiffman *et al* 12			✓	✓					
Vogt *et al* 26*			✓						
Vogt *et al* 43†			✓						✓
Borland *et al* 44				✓					
Willems *et al* 45	✓		✓	✓	✓				✓
Cobb *et al* 46									✓
Cook-Shimanek *et al* 30	✓	✓	✓	✓	✓	✓	✓		
Qualitative									
Vogt *et al* 26*	✓	✓	✓	✓	✓	✓		✓	
Vogt *et al* 43†			✓						✓
Uppal *et al* 34			✓	✓	✓			✓	
Data from current study	✓		✓	✓	✓	✓	✓	✓	✓

*Vogt *et al* 26 reported data from two studies, one qualitative and one quantitative

†Vogt *et al*^43^ reported data from two studies, one qualitative and one quantitative.

GP, general practitioner; NRT, nicotine replacement therapy.

Our central analytical focus, however, was the original, previously unreported categories in our analysis (table 3). When grouped, these suggested 4 new processes that could help explain unassisted quitting:

1. Prioritising lay knowledge;
2. Evaluating assistance against unassisted quitting;
3. Believing quitting is their personal responsibility;
4. Perceiving quitting unassisted to be the ‘right’ or ‘better’ choice.

**Table 3:** The four analytical categories that explain the process and meaning of quitting unassisted, with illustrative quotes

Category	Participant quotes
Prioritising lay knowledge ▸ Valuing personal experiences ▸ Being influenced by shared/collective knowledge	‘*I've done this, I've done the gum before, it's my turn to just do it by myself with common sense and willpower*.’ Female, 57 years old ‘*I've known a couple of people around town that have tried to give up with patches and that and they've gone 3 or 4 weeks and they've started smoking again and all that*.’ Female, 52 years old ‘*I've got friends that have used the patches and the gum a lot. They've been unsuccessful. They've done the gum and the patches, I don't know how many times. They've spent so much money on them, and they just cannot make it work.*’ Female, 31 years old ‘*Well [assistance] hadn't worked in the past and I didn't think—I'd come to the realisation that it was just in the mind, it was just a matter of willpower, it was just a matter of saying no and sticking to it.*’ Male, 59 years old
Evaluating assistance against unassisted quitting ▸ Weighing up the ‘value’ assistance brings to them and their quit attempt (is it worth using assistance to quit?) ▸ Wanting to save money *now* (spending money to quit is irrational, especially on something that brings no ‘pleasure’) ▸ Wanting to quit ‘instantly’, be a non-smoker *now* (which assistance does not allow) ▸ Disliking the ‘inconvenience’ of assistance (assistance is too complicated, too fiddly) ▸ Associating assistance with additional effort (eg, adopting new, but temporary, routines)	‘*It was a big thing that if I'm going to save money by not smoking then why should I spend money on not smoking.*’ Male, 45 years old ‘*The cigarettes, that's the fun. Why would you spend $20 on non-fun?* ’ Female, 34 years old ‘*I found [NRT] expensive. I thought that if you're going to get nicotine anyway at least there should be some positive reason for it.*’ Female, 56 years old ‘*If I'm going to quit smoking I'm going to do it cold turkey and get it over and done with.*’ Female, 52 years old ‘*I went to the GP and he said oh, you need to continue to smoke though for a couple of—what was it? It is a week? I was like oh no, but I want to stop now.*’ Female, 34 years old ‘*It's too much of a hassle… You've got to go out and buy the thing. You've got to stick it on or chew it or unwrap it.*’ Male, 61 years old
Believing quitting is their personal responsibility ▸ Smoking and quitting are personal problems (and the responsibility of the individual) ▸ Smoking and quitting are not medical conditions ▸ The smoker is best placed to know how to quit, what will work	‘*It's my problem. Not problem, I think that's a bad choice of words, but I was the one smoking.*’ Male, 28 years old ‘*That's so important that you don't make an issue out of it. It is a personal—you're right. You are so right. It is a personal thing.*’ Male, 61 years old ‘*Yeah, okay, I screwed up, I smoked for years, I really need to do something about this and cope with it.*’ Female, 57 years old ‘*I'm not much of someone to go to a doctor unless there was, unless I thought there was a serious problem with myself I don't normally go to a doctor.*’ Male, 45 years old ‘*I'm independent and I'm stubborn and that's the only way that I knew how to do it. I wasn't going to—I'm not a person to ask for help. So I don't think I would have asked for help to quit smoking.*‘ Female, 31 years old ‘*OK I did the Champix, I stopped for maybe—I can't remember if it was 2 or 3 months—but like it didn't work because it actually, the change sort of wasn't from within,*’ Female, 56 years old ‘*I think quitting cold turkey, you're going to have more chance of actually [staying] a non-smoker, if you quit cold turkey....because I think that you need that willpower to stay motivated to not smoke.*’ Female, 31 years old ‘*Because grand scheme of things, it's always your willpower that's going to stop you. So you might be able to use other methods to help you quit smoking, but six months down the track, you need to have that willpower to stop you doing that again*.’ Female, 31 years old ‘*I feel a sense of accomplishment in knowing that I did it cold turkey. Knowing that I didn't have to go to other means to do it. That I was able to use my willpower*.’ Female, 31 years old
Perceiving quitting unassisted to be the ‘right’ or ‘better’ choice ▸ Quitting unassisted is the ‘best’ way to quit ▸ Equating quitting unassisted with being serious about quitting	‘*I think I just didn't want to [use assistance], I just felt that for me to do it properly I actually had to be able to do it myself.*’ Female, 50 years old ‘*[Taking medication] had crossed my mind, but I'm a fairly stubborn person I suppose. I don't really—I believe that I should be able to do it myself, without those sorts of things.*’ Male, 31 years old ‘*I think that if you're truly committed you don't need anything* ’ Female, 56 years old

Illustrative quotes for each category are provided in table 3.

### Prioritising lay knowledge

Many participants expressed views about assistance that were at odds with accepted knowledge in smoking cessation on the effectiveness, side effects and long-term safety of assistance (table 2). These ‘misperceptions’ about assistance appear to arise because participants’ personal experiences and lay knowledge of assistance do not tally with what they have been told about assistance by their general practitioner (GP), pharmacist or through direct-to-consumer marketing of NRT by pharmaceutical companies. The gulf between what smokers have personally experienced or heard from others, and what health professionals are telling them was particularly evident in participants’ talk of unmet expectations of what assistance could realistically do for them. For many, the experience of using assistance had not been as expected, including not being as effective as they had believed it would be.

Participants talked of the importance of shared narratives of assistance that were predominantly negative and shared narratives of quitting unassisted that were predominantly positive. Shared stories of assistance—both personal and secondhand—were stories of failure to quit, and of unpleasant and sometimes serious side effects. In contrast, talk about quitting unassisted often featured family and friends who had managed to quit successfully on their own.

In order to resolve the tension between what is going on in ‘their world’ and what the professional medical and healthcare worlds are endorsing, participants prioritised what they knew: either directly from their own experiences or indirectly from ‘trusted’ sources. As a consequence, participants appeared to discount professional advice in favour of their own first-hand quitting experiences and the collective narratives of quitting successes and failures that circulated in their social groups. This lay knowledge-making based on personal and collective experiences appears to be a powerful force at play in smokers’ decisions about quitting.

### Evaluating assistance against unassisted quitting

On the whole, participants did not seem to be quitting unassisted because of a lack of awareness or knowledge about the assistance available to them. Instead participants appeared to have engaged in an evaluation of the perceived costs and benefits of using assistance compared with the costs and benefits of quitting unassisted. Factors in this cost–benefit balance related primarily to the perceived convenience of unassisted quitting (in terms of time to being ‘quit’ and the effort required to make the quit attempt happen) and the importance of short-term financial savings. These arguments were sometimes explicit and sometimes implicit.

Participants talked about wanting to quit now, immediately. NRT and smoking cessation medication both involve a treatment period in which the smoker is still a smoker: they cannot yet call themselves a ‘non-smoker’. In their opinion, use of assistance essentially delays their progression to being totally quit. In contrast, going ‘cold turkey’ (ie, quitting suddenly without cutting down or using any assistance) provides an immediate satisfaction and instant non-smoker status. There often appeared to be a sense of urgency or a need for an immediate and complete change of status in those who opted to quit unassisted.

Using assistance was also associated with an investment of practical and logistical effort. Assistance required the adoption of new—but temporary—routines and habits. It was a middle ground or half-way house through which the smoker would have to pass. They would have to complete this ‘assistance’ phase before being able to adopt yet another set of routines and habits to become nicotine-free or drug-free. These temporary routines associated with assistance included obtaining or purchasing assistance, carrying it around and remembering to use it. For some this temporary, additional set of routines appeared simply too complex, too bothersome and too high a price to pay in terms of the inconvenience generated.

For a number of participants, spending money to quit, especially when quitting was motivated by a desire to save money, appeared counter-intuitive. For such participants, thoughts were focused on the here and now, on the short-term rather than long-term savings. Few participants appeared to regard money spent on assistance as a long-term investment in future financial savings. As a consequence, using assistance to quit was viewed as a barrier to maximising potential savings while quitting. For NRT specifically, this balancing of the pros and cons extended beyond the financial cost of cigarettes versus cost of NRT to the perceived pleasure that the financial spend was likely to provide. Spending $20 on cigarettes was reasonable because it would deliver pleasure; spending $20 on something that was going to make you miserable was not. An unwillingness to spend on NRT also appeared related to an inability to reconcile nicotine's dual role as part of the problem and the solution, and to fears of becoming addicted to NRT gums, patches or inhalers.

### Believing quitting is their personal responsibility

Quitting appeared to be an intensely individual experience and one that the smoker believed only they could take charge of. Ultimately quitting was something they had to face themselves. Many participants seemed to have reached a point where they regarded smoking to be their problem and quitting to be their personal responsibility. Quitting was, therefore, not necessarily something that could be helped or facilitated by external support (be it from family, friends or health professionals).

Participants often talked about being the person best placed to know why they smoked, why they wanted to quit, and what was likely to work for them. To these participants, external help or assistance was unlikely to be useful or necessary. For many this appeared to be because they had previous experience of unsuccessful assisted quit attempts (with, eg, over-the-counter NRT, prescription NRT, smoking cessation medications or behavioural support) and had learnt that for them, assistance was unhelpful or solved only part of the problem. Conversely, other participants had not previously used professional or pharmacological support to quit and therefore, did not see the need to do so now. Others simply did not equate smoking with being ill, or regard smoking and quitting as medical conditions: this meant medical support was not appropriate and little benefit would be gained from involving a GP in the quit attempt. Several participants implied that a GP would be able to offer only generic or lay quitting advice that was unlikely to be relevant to them personally: in other words, from the participant's perspective, the GP could add little to the participant's own personal store of quitting experiences.

A number of participants also appeared to have an issue with adopting a substitute behaviour (ie, NRT or smoking cessation medication). To these participants, the use of NRT or drugs meant that they were still dependent on nicotine or another substance to deal with their need for nicotine. If they really wanted to quit and to quit for good, they needed to take that step themselves, which to them essentially precluded use of assistance and in particular, NRT.

### Perceiving quitting unassisted to be the ‘right’ or ‘better’ choice

In contrast to the dominant medical and health promotion discourse about quitting unassisted being undesirable or even foolhardy, for many participants quitting unassisted was the ‘right’ or ‘better’ way to quit. This belief appeared to be closely associated with what participants referred to as ‘being serious’ about quitting. It appears that underlying these beliefs may be a set of values that the participant and perhaps also Australian society, as a whole, endorses.

Participants talked, either explicitly or implicitly, about the values that were important to them in relation to their quit attempt: independence, strength, autonomy, self-control and self-reliance. These values are, broadly speaking, also reflective of values central in many western societies and cultures. It seems likely that these broadly held values were influential in shaping participants’ beliefs about quitting unassisted being the right or better choice and the belief that quitting was ‘up to me’. Quitting unassisted allowed the participant to realise a need to feel independent, in control and autonomous, something that they would not necessarily have felt if they had used assistance. Some participants even suggested that seeking help from a GP or another source such as the Quitline would be tantamount to admitting failure. The independent nature of their quit attempt was seen as an important contributor to the success of that attempt.

In summary, many participants believed they had achieved something of value by quitting unassisted, and appeared to take this achievement as an indicator of the strength of their moral character. In this context, quitting unassisted was presented as a morally superior option; quitting unassisted was evidence of personal virtue. It is important to note, however, that this was rarely used as a measure of the moral worth of others. Participants rarely suggested that other smokers who used assistance to quit were morally inferior. Rather, they presented their final, unassisted quit attempt as evidence that their personal virtue had increased over time, thus bolstering their own sense of identity and self-worth.

## Discussion

### Principal findings

In this community sample of ex-smokers who had quit on their own without consulting their GP or using smoking cessation assistance, issues of cost and access to assistance, misperceptions relating to the effectiveness and safety of pharmacotherapy, and confidence in their ability to quit on their own affected their decision to quit unassisted. This was consistent with earlier quantitative and qualitative research (table 2). However, we found that the influences on non-use of assistance were more complex, involving careful judgements about the value of knowledge, the value of different quitting strategies, the importance of taking personal responsibility and the moral significance of quitting alone. Future efforts to improve uptake of assistance may need to take some of these influences into consideration.

In an effort to understand what appears to be conflicting advice about quitting and how to quit successfully, participants appear to fall back on trusting their intuition or common sense, giving preference to their personal and shared knowledge of quitting over professional or theoretical knowledge. Lay knowledge (or lay epidemiology) has previously been used to understand how health inequalities develop in smokers,^47–49^ to inform health-promotion practices in smoking cessation,^50^ and to explain the range of self-exempting beliefs used by smokers to avoid quitting.^51^ Our study is the first to demonstrate how lay knowledge influences non-use of assistance when attempting to quit smoking.

Participants who quit on their own often appeared reluctant to consult their GP, primarily because they did not view smoking or quitting as an illness, reflecting what others have also reported.^52^ ^53^ Our analyses show that this reluctance to consult a GP may also be because smokers perceive the GP has little to offer beyond the smoker's own lay knowledge, reflecting what others have recently reported for smoking cessation consultations in general practice in the UK.^54^ This reluctance to consult a GP may be reinforced if the smoker is hesitant about using pharmacotherapy or if they believe smoking is not a ‘doctorable’ condition. Doctorable is a term coined by Heritage and Robinson^55^ to explain the way in which patients in the USA account for their visits to primary care physicians and to demonstrate how patients orientate to a need to present their concerns as doctorable. Before visiting a physician, patients make a judgement as to whether they require medical help. They are aware that the physician will subsequently judge their judgement when they present at the surgery. It is conceivable that this need to present only when the individual perceives the condition to be doctorable could apply not just to smoking cessation, but to other difficult-to-change health behaviours such as losing weight or getting fit.

In addition to judgements relating to the value of lay knowledge, our study highlights how smokers make judgements about the value of different quitting strategies based on perceptions of time and effort required, convenience and cost. This process of evaluation has been reported for decisions related to the taking of other prescribed medications.^56^ Pound *et al* 56 reported that patients often weigh-up the benefits of taking a medicine against the costs of doing so and are often driven by an overarching desire to minimise medicine intake. In the current study, this evaluation of different quitting strategies often resulted in the participant forming a negative opinion of assistance and in particular, of NRT. Given nicotine's complicated history and transformation from an addictive, toxic and potentially harmful drug to a medically useful drug it was not surprising that many participants found it difficult to reconcile nicotine's portrayal as being part of the problem and a possible solution,^57^ and as a result appeared to be resisting use of medications to assist them to quit.

Layered underneath the prioritising of lay knowledge and the evaluation of different quitting strategies were deep-rooted cultural values, such as independence, strength, self-reliance, self-control and autonomy, which influenced participants’ views on assisted and unassisted quitting. Lay knowledge in combination with these multilayered influences lead many participants to believe that quitting unassisted was the ‘right’ or ‘better’ way to quit, that the participant was personally responsible for their quitting and that quitting unassisted was a prerequisite for ‘being serious’ about quitting. This key concept, being serious, is one we believe is critically important to Australian smokers and one we are exploring further in our ongoing research.

It should be noted that this study included only successful ex-smokers (quit for at least 6 months). Given that these individuals were interviewed in the context of a successful quit attempt, attribution theory^58^ might provide some insight into the emergence of independence, strength, self-control and personal virtue as components of the successful unassisted quit attempt in these interviews. Attribution theory suggests a self-serving bias in attributions such that success is attributed to internal factors (such as personal virtue), and failure to external or situational factors. It might be informative to conduct some research with smokers who tried to quit on their own and failed, as well as with ex-smokers who successfully quit with assistance to explore whether concepts relating to external or internal attributions emerge for these different groups of quitters.

### Strengths and limitations

The qualitative design and in particular, the grounded theory methodology is a strength of this exploratory study. The concurrent data collection and analysis allowed unanticipated findings to emerge (such as the importance of lay knowledge and the sense of the participant being personally responsible for their quitting) and to be followed up and more fully explored in subsequent interviews. Allowing ex-smokers to talk about previous assisted and unassisted quit attempts provided new insights into why smokers quit unassisted. The qualitative design of the current study allowed us to extend the existing literature on barriers and facilitators of assistance utilisation to provide a more in-depth discussion of the complex reasons for why many smokers may choose to quit unassisted. By using a sample of ex-smokers from the general population we were able to broaden previous research that had focused specifically on at-risk or disadvantaged subpopulations.^31–33^ In the current study, rather than controlling for context, we actively sought to retain context in order to reveal the historical, social and cultural factors that may have impacted on quitting decisions. Limitations of the current study include using a non-representative sample of ex-smokers. Nonetheless, we minimised volunteer bias by recruiting directly from the general community. By recruiting through mainstream (press releases, newspaper articles and talkback radio) and social media (Twitter and Facebook), screening potential participants and providing participants with financial reimbursement for sparing their time, we achieved a sample of ex-smokers from diverse socioeconomic backgrounds who varied in age, education, income, geographical location, prior quitting experiences and prior use of assistance. As participants were ex-smokers who had quit unassisted between 6 months and 2 years ago, it is possible that their recollections may have been subject to recall bias. However, this possibility was balanced against the potential for relapse to smoking, which was an important consideration for this study.

### Implications and future research

A proportion of smokers are unlikely to choose to use assistance to quit smoking or are reluctant to do so. Too much focus on pharmacological assistance may fail this group. It may be a more productive and a potentially more patient-centred approach to acknowledge that for these smokers quitting unassisted is a valid and potentially effective option.

Evidence-based medicine (EBM) and clinical practice guidelines prioritise results from randomised controlled trials (RCTs) and meta-analyses of RCTs. As a consequence, current smoking cessation guidelines in the UK,^18^ USA^19^ and Australia^20^ position pharmacotherapy as first-line therapy for those dependent on nicotine (>10 cigarettes per day). A range of government policies ensure pharmacotherapy is free or heavily subsidised, available on prescription and/or over-the-counter and that smokers have access to widely promoted and free quitline advice and support, and/or dedicated stop-smoking services.

As RCTs are designed to evaluate the efficacy of interventions, such as medications, in carefully controlled study populations, they cannot capture and often seek to eliminate the complexities associated with patients’ lived experiences. This complexity may, however, be of relevance when making decisions about how to manage patients with complicated health-related behaviours, such as smoking. By retaining and examining some of the complexity surrounding quitting smoking, we have highlighted how participant's beliefs, values and preferences can influence the decision to quit unassisted. Previous research into patient-centred care has also identified that respect for a patient's beliefs and values,^59^ needs and preferences,^60^ and knowledge and experience^61^ are central to delivering care that is tailored to the needs of the individual patient. Accordingly, patient-centred care for smokers may include recognising and respecting smokers’ reasons for declining assistance, validating and approving their choices and modifying brief interventions to support their preference for unassisted quitting, where preferred.

Healthcare policy does not operate in a vacuum. As our study indicates, success of any given policy is critically dependent on the broader social and cultural context. This is especially true for tobacco control given the influence of key stakeholders such as the tobacco industry. Recent research highlights how the tobacco industry capitalised on the powerful notion of personal responsibility to frame tobacco problems as a matter for individuals to solve.^62^ To our knowledge, our analysis is the first to indicate smokers do indeed feel personally responsible for quitting. Smoker's beliefs about quitting have been heavily influenced by social and cultural ideals, some of which are highly likely to have been shaped by the tobacco industry's individual choice rhetoric. The complexity of how such a rhetoric has influenced smokers has to date been unexplored.

The value placed on lay knowledge and on different quitting strategies by participants indicates that GPs, health promotion practitioners and pharmaceutical companies may be advised to be mindful of the consequences of overselling assistance and potentially unrealistically raising smokers’ outcome expectations, further fuelling the apparent gulf between lay experiences and expert-derived knowledge. The low absolute efficacy rates of NRT and stop-smoking medications^1^ create a challenge: is it possible to communicate about these products without disheartening smokers or making promises that may be difficult to deliver?

Cultural values are likely to play a role in the choice to use assistance or not, and future research should explore these issues in other cultures. It would be useful to replicate this study in other cultural contexts and in countries less advanced in tobacco control to determine whether the study findings are applicable across countries, cultural dimensions and stages of the tobacco epidemic.

For those patients who do seek medical advice, GPs may need to be cognisant of the role of lay knowledge and the patient's evaluation of different quitting strategies when counselling and advising about quitting smoking. The challenge will be to support those smokers who wish to quit unassisted while avoiding stigmatisation of those smokers who want or need assistance to quit.

## Conclusion

A smoker’s reluctance to use assistance to quit may sometimes be difficult to understand. Through this empirical work we are now able to suggest some explanations for this behaviour.

The reasons for smokers’ rejection of the conventional medical model for smoking cessation are complex and go beyond the modifiable or correctable issues relating to misperceptions or treatment barriers. Lay knowledge and contextual factors are critically important to a smoker's decision to seek or resist assistance to quit. Smokers prioritise lay knowledge, evaluate assistance against unassisted quitting, believe quitting is their personal responsibility and perceive quitting unassisted to be the right or better option. Accordingly, GPs might recognise and respect smokers’ reasons for rejecting assistance, validate and approve their choices, and modify brief interventions to support their preference for unassisted quitting, where preferred.
